# Reaffirmation of known major genes and the identification of novel candidate genes associated with carcass-related metrics based on whole genome sequence within a large multi-breed cattle population

**DOI:** 10.1186/s12864-019-6071-9

**Published:** 2019-09-18

**Authors:** D. C. Purfield, R. D. Evans, D. P. Berry

**Affiliations:** 1 Animal & Grassland Research and Innovation Center, Teagasc, Moorepark, Fermoy, Co. Cork Ireland; 2 Irish Cattle Breeding Federation, Bandon, Co. Cork Ireland

**Keywords:** Carcass weight, Conformation, Fat, Beef, GWAS, QTL, Bovine, Myostatin

## Abstract

**Background:**

The high narrow sense heritability of carcass traits suggests that the underlying additive genetic potential of an individual should be strongly correlated with both animal carcass quality and quantity, and therefore, by extension, carcass value. Therefore, the objective of the present study was to detect genomic regions associated with three carcass traits, namely carcass weight, conformation and fat cover, using imputed whole genome sequence in 28,470 dairy and beef sires from six breeds with a total of 2,199,926 phenotyped progeny.

**Results:**

Major genes previously associated with carcass performance were identified, as well as several putative novel candidate genes that likely operate both within and across breeds. The role of *MSTN* in carcass performance was re-affirmed with the segregating Q204X mutation explaining 1.21, 1.11 and 5.95% of the genetic variance in carcass weight, fat and conformation, respectively in the Charolais population. In addition, a genomic region on BTA6 encompassing the *NCAPG/LCORL* locus, which is a known candidate locus associated with body size, was associated with carcass weight in Angus, Charolais and Limousin. Novel candidate genes identified included *ZFAT* in Angus, and *SLC40A1* and the olfactory gene cluster on BTA15 in Charolais. Although the majority of associations were breed specific, associations that operated across breeds included *SORCS1* on BTA26, *MCTP2* on BTA21 and *ARL15* on BTA20; these are of particular interest due to their potential informativeness in across-breed genomic evaluations. Genomic regions affecting all three carcass traits were identified in each of the breeds, although these were mainly concentrated on BTA2 and BTA6, surrounding *MSTN* and *NCAPG/LCORL*, respectively. This suggests that although major genes may be associated with all three carcass traits, the majority of genes containing significant variants (unadjusted *p*-value < 10^− 4^) may be trait specific associations of small effect.

**Conclusions:**

Although plausible novel candidate genes were identified, the proportion of variance explained by these candidates was minimal thus reaffirming that while carcass performance may be affected by major genes in the form of *MSTN* and *NCAPG/LCORL*, the majority of variance is attributed to the additive (and possibly multiplicative) effect of many polymorphisms of small effect.

**Electronic supplementary material:**

The online version of this article (10.1186/s12864-019-6071-9) contains supplementary material, which is available to authorized users.

## Introduction

Profit in cattle production systems is a function of both revenue and costs of production. In beef cattle finishing systems, carcass value is the main revenue source which is dictated by both quantity (i.e., weight) and quality (e.g. proportion of carcass as high value cuts, eating quality). The eventual carcass phenotype realised (as with any phenotype) is a function of both the underlying genetic potential of the animal and the environment the animal has been exposed to. The high narrow sense heritability of carcass traits in cattle [1–3] suggests that the underlying additive genetic potential of an individual should be strongly correlated with both animal carcass quality and quantity, and therefore, by extension, carcass value. Hence, dissecting the genomic architecture governing carcass merit has multiple uses, not least, by contributing to potentially accelerated genetic gain via more accurate estimation of the additive genetic merit of individual animals. Other uses include benchmarking of herds through the comparison of actual phenotypic carcass merit with expectations based on additive genetic merit, but also by informing nutritional and management strategies to maximise carcass value by means of a deeper understanding of the biological pathways supporting carcass growth.

Despite the vast quantity of phenotypic cattle carcass data available, studies have identified relatively few quantitative trait loci (QTL) associated with carcass performance. Genomic regions on *Bos Taurus* autosomes (BTA) 6 and 14, flanking the *LCORL* and *PLAG1* genes, respectively, have been putatively associated with carcass weight in both dairy and beef cattle breeds [4–6]. Similarly, regions on BTA14 and on both BTA10 and 29 have been associated with carcass fat and conformation, respectively in cattle [7–10]. To our knowledge, however, few studies have compared QTL for carcass traits in both dairy and beef breeds, and even fewer such studies have been undertaken using (imputed) whole genome sequence [10, 11]. Association studies completed with imputed sequence variants have been successful in pinpointing candidate causal variants that control complex trait variation (cattle stature; [12]; cattle milk fat and protein; [13]).

The objective of the present study was to detect genomic regions associated with three carcass traits, namely carcass weight, conformation and fat cover, using imputed whole genome sequence data in 28,470 dairy and beef sires with phenotyped progeny; carcasses were assessed for conformation and fat score based on video image analyses. Detected genomic regions associated with each of the three carcass traits was compared within and across breeds. In addition, genomic regions associated with all three carcass traits were also identified.

## Results

Genotypes of 41,389,526 sequence SNPs were imputed for 28,470 sires from six cattle breeds with a combined 2,199,926 progeny, and used to identify genomic regions associated with carcass-related metrics within and across breeds. The breeds represented included Angus (AA; *n* = 2366), Charolais (CH; *n* = 11,219), Hereford (HE = 1216), Holstein-Friesian (HF; *n* = 2372), Limousin (LM; *n* = 9747) and Simmental (SI; *n* = 1550). De-regressed estimated breeding values for three carcass traits were analysed including carcass weight, carcass fat and carcass conformation and genomic regions associated with all three traits were also examined. Strong regions of known association were detected for all three carcass traits in the more numerous breed populations and several putative novel candidate genes were also suggested.

### Within-breed associations

**Fig. 1:**
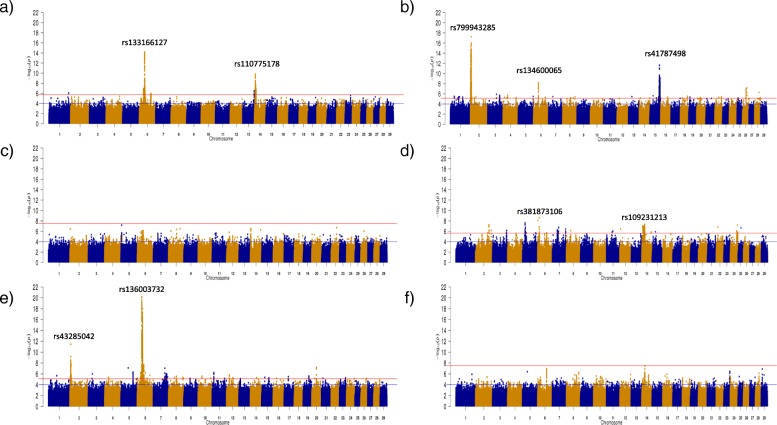
Manhattan plots for carcass weight in each of the six breeds: **a**) Angus **b**) Charolais **c**) Hereford **d**) Holstein-Friesian **e**) Limousin and **f**) Simmental. The red line indicates the Benjamini and Hochberg significance threshold within each breed and the blue line is a *p*-value threshold of 10^− 4^

Across all three carcass traits analysed, no SNPs remained significant after adjustment for Benjamini and Hochberg multiple testing with a false discovery rate of 5%, in either the HE or SI populations. QTLs were defined as all regions where a minimum of three significantly Benjamini and Hochberg p-adjusted SNPs resided within 500 kb of each other. In total 618, 2617, 682, and 2849 SNPs were associated (adjusted *p* < 0.05) with carcass weight within the AA, CH, HF and LM populations (Fig. 1) and a total of 9, 15, 20 and 20 QTL were subsequently identified within each breed, respectively (see Additional file 1).

**Fig. 2:**
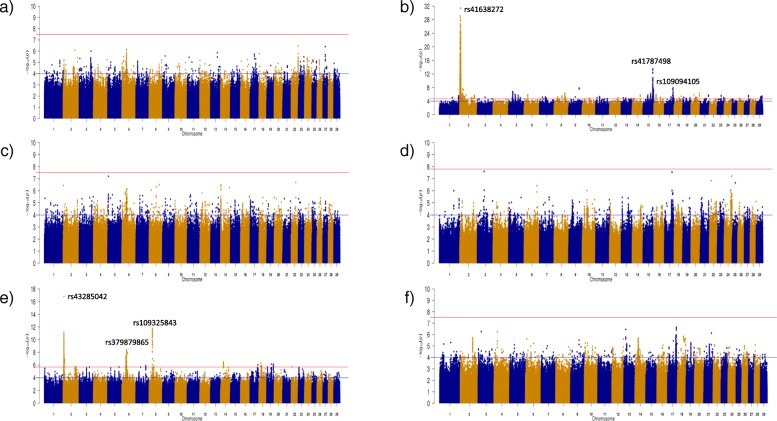
Manhattan plots for carcass fat in each of the six breeds: **a**) Angus **b**) Charolais **c**) Hereford **d**) Holstein-Friesian **e**) Limousin and **f**) Simmental. The red line indicates the Benjamini and Hochberg significance threshold within each breed and the blue line is a p-value threshold of 10^− 4^

A total of 8229 SNPs in the CH population and 731 SNPs in the LM population were associated with carcass fat; no SNP in any of the other breeds was associated with carcass fat (Fig. 2). The majority (i.e., 82.42%) of the SNP associations with carcass fat in the CH population were located on BTA2 and these could be collapsed into 9 distinct QTL (Additional file 2). Similarly, 70.18% of the SNP associations with carcass fat in the LM population were also located on BTA2 but in just two QTL regions (see Additional file 2).

**Fig. 3:**
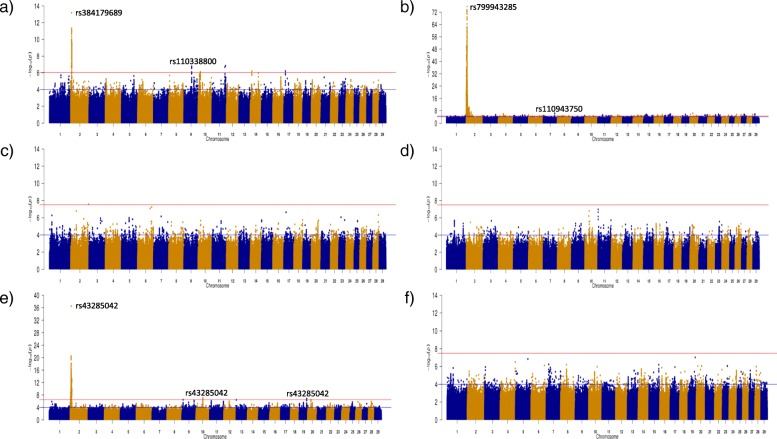
Manhattan plots for carcass conformation in each of the six breeds: **a**) Angus **b**) Charolais **c**) Hereford **d**) Holstein-Friesian **e**) Limousin and **f**) Simmental. The red line indicates the Benjamini and Hochberg significance threshold within each breed and the blue line is a p-value threshold of 10^− 4^

For carcass conformation, several genomic regions were identified within the AA, CH and LM populations (Fig. 3). The CH population had the largest number of significant SNPs with a total of 17,900 SNPs remaining significant after adjustment for multiple testing, whereas 321 and 2114 significant SNPs were associated with carcass conformation in the AA and LM populations, respectively. Thirty-two QTL regions across 16 different chromosomes were identified in the CH population, whereas 2 and 12 QTLs were identified in the AA and LM populations, respectively (see Additional file 3).

The strongest SNP association detected within a breed was often the strongest association across two or all of the carcass traits. For example, in the CH population, the same SNP, rs799943285 an intergenic variant on BTA2, was the SNP with the strongest association with carcass weight (unadjusted *p* = 5.92 × 10^− 18^) and carcass conformation (unadjusted *p* = 1.60 × 10^− 76^), explaining 1.21 and 5.95% of the genetic variation, respectively. However, the strongest association with carcass fat in the CH breed, rs41638272, was located 107 kb further upstream on BTA2. The QTLs containing each of these SNPs overlapped on BTA2 from 1.851 to 8.394 Mb and encompassed a total of 40 genes including *MSTN*. This genomic region on BTA2 was also significantly associated with each of the carcass traits in the LM population. The SNP, rs43285042 on BTA2, had the strongest SNP association within this QTL for each of the carcass traits in the LM but the allele substitution effect direction was opposite for carcass fat relative to both carcass weight and conformation. The proportion of genetic variance explained by rs43285042 in the LM population ranged from 1.34% for carcass weight to 4.35% for carcass conformation. Although BTA2 was strongly associated with each of the carcass traits in the LM population, it was BTA6 that contained the strongest associations with carcass weight in the LM population. In fact, 84.69% of the 2849 significant SNPs associated with carcass weight in the LM population were located on BTA6. These significant SNPs were primarily distributed across two QTL on BTA6; the first was from 32.210 to 33.884 Mb encompassing five uncharacterised genes and the second was further downstream from 37.463 to 42.711 Mb surrounding 22 genes including the *LCORL/NCAPG* locus. The latter QTL also overlapped significant associations detected in the AA and CH populations for carcass weight. Within the HF population, only QTL associated with carcass weight were identified. The strongest association in the HF was a downstream variant of *PLAG1* (unadjusted *p* = 4.54 × 10^− 8^). In comparison to the other breeds, the positive alleles identified with the HF population were almost fixed in the majority of the QTLs; the most significant SNP within 16 of the 20 QTLs associated with carcass weight had a positive allele frequency > 0.99.

Genome annotation revealed multiple missense variants were significantly associated with all three carcass traits. A total of 3 significant missense variants were associated with carcass weight in the AA population, representing a 1.85 fold enrichment of missense variants among the significant variants as compared to what would be expected by chance (Table 1). One of the missense variants within *TMPRSS11A*, rs452419999, was determined to be deleterious on protein function with a sorting intolerant from tolerant (SIFT) score of zero and was located in exon 2 in the transmembrane helix region where a leucine amino acid becomes replaced with a phenyalanine amino acid. The allele frequency of the positive allele (A), which was predicted to be deleterious according to the SIFT scoring system, was 2.07% within the AA population; a similar allele frequency was detected in the HF population (4.09%) whereas the A allele was only marginally segregating in the remaining beef breeds (< 1%). Of the 10 missense variants significantly associated with carcass weight in the CH population, all were located on BTA15 but only one, rs210125929 in the olfactory receptor *OR5AK2*, was deemed to be deleterious on protein function (SIFT score 0.01). The remaining 9 significant missense variants were located in either *ENSBTAG00000014309* or *ENSBTAG00000039331*, both of which are olfactory receptor orthologues. Similar to carcass weight, variants within olfactory genes on BTA15 were also significantly associated with carcass fat in the CH population; the missense SNP, rs446111343, located in *ENSBTAG00000038539* was strongly associated with carcass fat (unadjusted *p* = 2.36 × 10^− 6^) and had a SIFT score of 0.01. The sole significant missense variant identified in the HF population located within *FBX032* was also deemed to be deleterious to protein function (SIFT score 0), although the frequency of the SIFT predicted deleterious allele (T) was low (0.27%) within the population.

**Table 1:** Fold enrichment or depletion for each annotation class for all variants significantly* associated with carcass merit

Trait	Breed	No Sig SNPs	SNP annotation										
			Intergenic	Upstream	Downstream	3′ UTR	5′ UTR	Intronic	Missense	Synonymous	Stop gained	Non coding transcript	Splice donor
Carcass weight	AA	618	1.15 (476)	0.49 (10)	0.56 (11)	0	8.38 (2)	0.72 (114)	1.85 (3)	0.89 (2)	0	0	0
	CH	2617	1.05 (1840)	1.1 (95)	1.59 (133)	3.61 (17)	0	0.73 (495)	1.46 (10)	1.79 (17)	14.72 (1)	11.53 (9)	0
	HF	682	1.16 (533)	0.77 (16)	1.3 (28)	0.84 (1)	0	0.58 (101)	0.58 (1)	0.85 (2)	0	0	0
	LM	2849	1.27 (2416)	0.71 (48)	0.69 (63)	1.55 (8)	1.76 (2)	0.41 (305)	0.13 (1)	0.39 (4)	12.86 (1)	1.17 (1)	0
Carcass fat	CH	8229	0.99 (5451)	0.81 (221)	1.14 (301)	2.43 (36)	0.31 (1)	1.02 (2155)	0.93 (20)	1.44 (42)	4.43 (1)	0	8.17 (1)
	LM	731	1.26 (612)	0.08 (2)	0.34 (8)	1.51 (2)	3.44 (1)	0.54 (103)	0.51 (1)	0.77 (2)	0	0	0
Carcass conformation	AA	321	1.41 (304)	0.09 (1)	0.19 (2)	1.75 (1)	0	0.13 (11)	2.37 (2)	0	0	0	0
	CH	17,900	1.09 (12974)	0.60 (358)	0.84 (482)	1.39 (45)	1.27 (9)	0.85 (3920)	0.83 (40)	1.09 (69)	2.04 (1)	0.38 (2)	0
	LM	2114	1.18 (1665)	0.24 (17)	0.59 (40)	1.56 (6)	1.19 (1)	0.67 (368)	1.23 (7)	1.17 (9)	17.23 (1)	0	0

*Significant *p* < 0.05 after Benjamini and Hochberg correction

The number of significant SNPs within each annotation class is in parenthesis

Although, the detected significant associations for carcass fat were not enriched for missense variants (Table 1), 20 missense variants in the CH population and one in the LM population were significantly associated with carcass fat. The significant missense variants within the CH population were primarily located in the QTL spanning from 0.007 to 10.095 Mb on BTA2; 16 significant missense variants were identified in this QTL and three were predicted to be deleterious, including rs110065568, the F94L mutation located within the *MSTN* gene. A similar trend was also detected for carcass conformation, where 39 of the significant missense variants within the CH population, and all of the significant missense variants in the LM population and AA population, were located on BTA2 in QTLs overlapping the *MSTN* gene. In addition, the stop gain variant rs110344317, also known as the Q204X mutation within the *MSTN* gene, was significantly associated with all the three carcass traits in the CH population and with carcass fat and conformation in the LM population (Table 1) although it was not the strongest association within this QTL.

### Across-breed associations

**Fig. 4:**
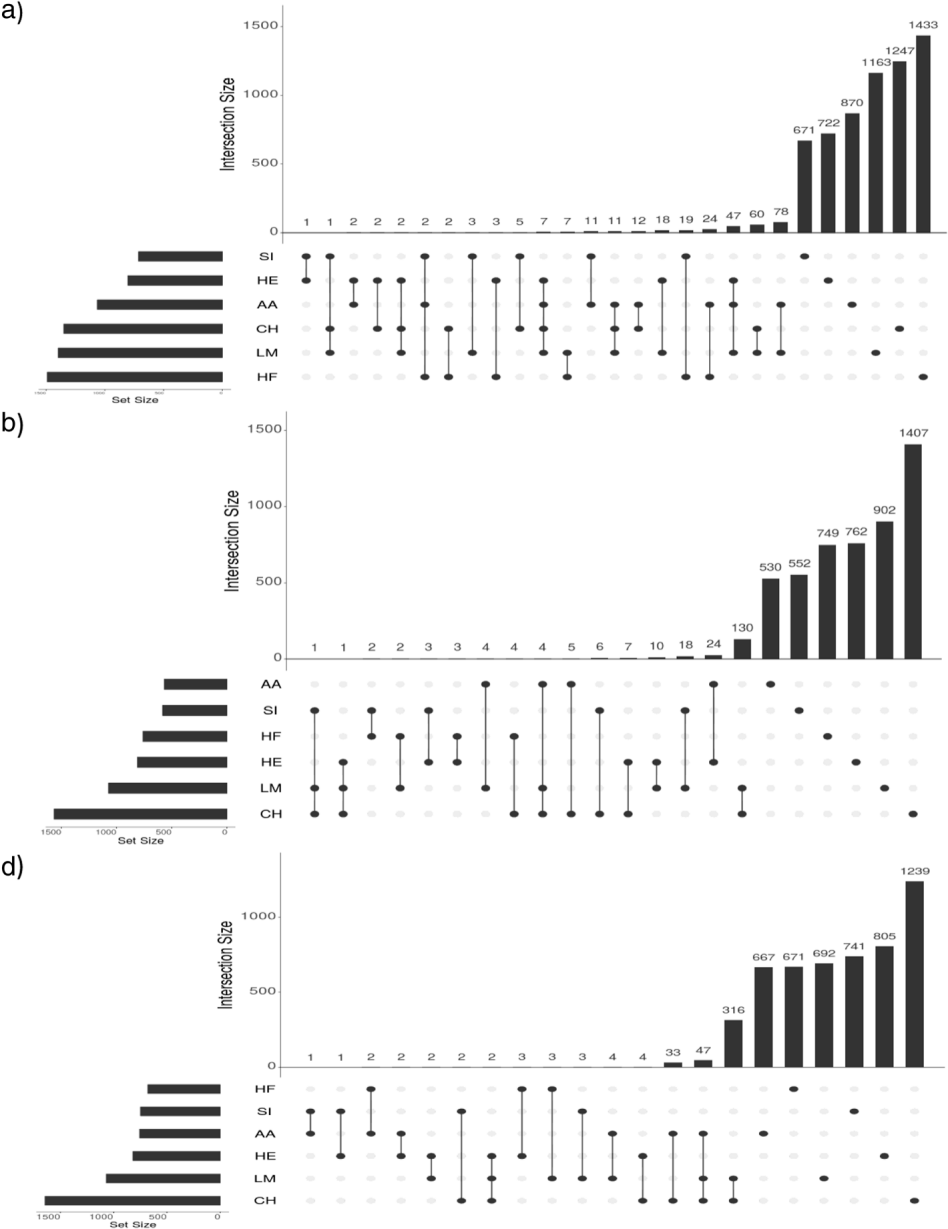
The number of 10 kb windows containing a SNP with an unadjusted p-value < 10^− 4^ across the genome that overlapped between different combinations of breeds including those unique to a given breed (far right)*. *Set size represents the number of windows in each breed containing a SNP with an unadjusted p-value < 10^− 4^ and intersection size is the number of such windows that were shared or unique across breeds. **a**) Signifies the number of windows for carcass weight, **b**) carcass fat and **c**) carcass conformation. AA represents Angus, CH represents Charolais, HE represents Hereford, HF represents Holstein-Friesian, LM represents Limousin and SI represents Simmental

Genomic regions associated with either of the carcass traits in more than one breed were identified using two approaches; 1) identifying overlapping 10 kb windows that contained at least one SNP with an unadjusted *p*-value < 10^− 4^ within each breed and 2) by undertaking a multi-breed genome-wide association across all 28,470 sires with breed fitted as a fixed effect. In the window-based analyses, the majority of the 10 kb windows harbouring a significant SNP (unadjusted p-value < 10^− 4^) were unique to a single breed and only a small proportion of overlap was evident in more than one breed; no window was significant in all six breeds for any of the traits (Fig. 4).

The CH and LM populations had the greatest number of overlapping windows (Fig. 4) with 316 windows common to both breeds identified on BTA2, 6 and 20 for carcass conformation. The majority (96.84%) of these windows were located on BTA2 between 0.58 to 10.39 Mb and encompassed the *MSTN* gene. Two genomic regions on BTA6, the first from 0.45 to 0.53 Mb and the second at 90.92 Mb surrounding the pseudogene *ENSBTAG00000032764* and *MTHFD2L,* respectively, and one region on BTA20 spanning from 24.95 to 29.97 Mb overlapping *ARL15* were identified from the remaining shared windows between the CH and LM populations. Overlap across four breeds (AA, HE, CH and LM) was identified on BTA6, where 7 windows spanning from 38.67 to 39.02 Mb encompassing the *NCAPG* and *LCORL* genes were significantly associated with carcass weight. Relative to carcass weight or carcass conformation, proportionally more significant windows were unique to each breed for carcass fat; on average, 92.48% of windows detected across all breeds harbouring significant SNPs for carcass fat were unique, compared to 89.57 and 87.41% for carcass weight and conformation, respectively. Across all breeds, the HF population had the greatest percentage of unique significant windows across all traits; on average across traits, 97.71% of all significant windows identified in the HF population were only significant in the HF population, whereas the LM population had the lowest percentage of unique significant windows; 77.37% of significant windows identified in the LM population were unique to the LM population.

**Fig. 5:**
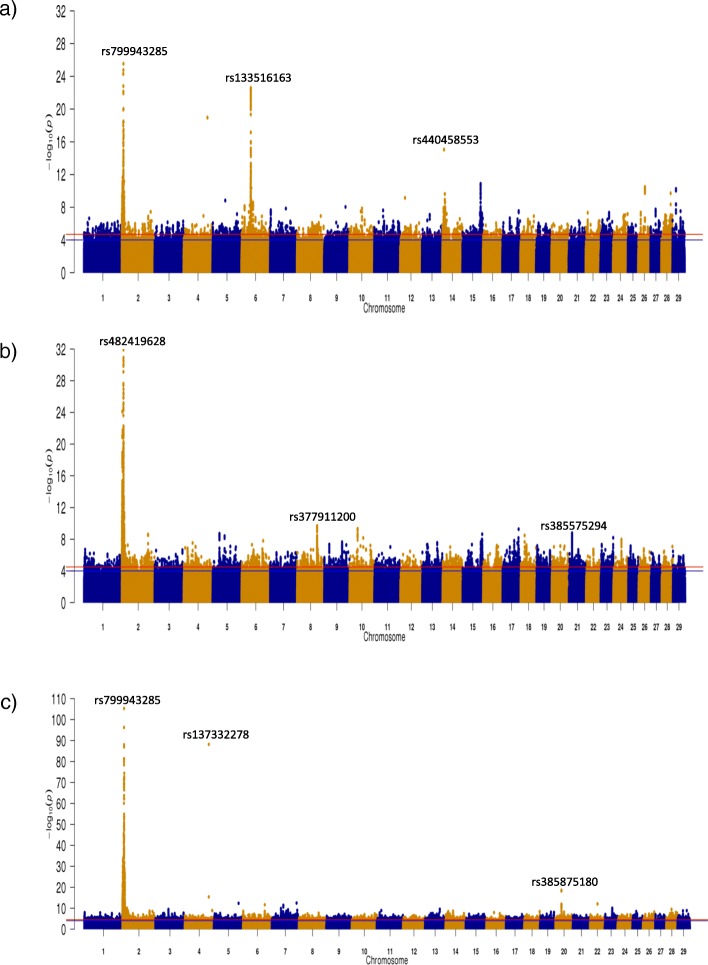
Multi-breed Manhattan plots for **a**) carcass weight **b**) carcass fat and **c**) carcass conformation across 28,470 sires with breed included as a fixed effect

Several strong associations were detected in the multi-breed analysis for each of the carcass traits, although the strongest association for each trait was a QTL encompassing the *MSTN* gene on BTA2 (Fig. 5). The *NCAPG/LCORL* locus, *ZFAT*, *PRDM11* and *SORCS1* genes which were all previously identified in the within breed analyses for carcass weight (see Additional file 1) were again identified in the multi-breed analysis, albeit with greater significance. *PTCH1* on BTA8 and the olfactory gene cluster on BTA15 which were associated with carcass fat in the CH population were also associated with carcass fat in the multi-breed analyses with greater significance. An additional advantage of completing the multi-breed analyses was that novel QTL not previously identified in the within breed analyses were identified; for example the QTL on BTA20 spanning from 21.525 to 27.054 Mb associated with carcass conformation. The strongest SNP association in this QTL on BTA20 was an intronic SNP (rs385875180) in *ARL15*. The lone SNP on BTA4 (rs137332278) strongly associated with both carcass weight and conformation was also a novel association and was located in the novel gene *ENSBTAG00000031548*, which is a member of the solute carrier family 23 vitamin C transporters. Similarly, a novel QTL on BTA21 spanning from 12.577 to 13.433 Mb encompassing the *MCTP2* gene was identified to be associated with carcass fat. Variants within *ARL15* and *MCTP2* were moderately segregating within each of the breeds suggesting scope for selection exists, although rs137332278 on BTA4 was found to be fixed for the positive G allele in AA, CH and LM populations.

### Associations with more than one carcass traits

**Fig. 6:**
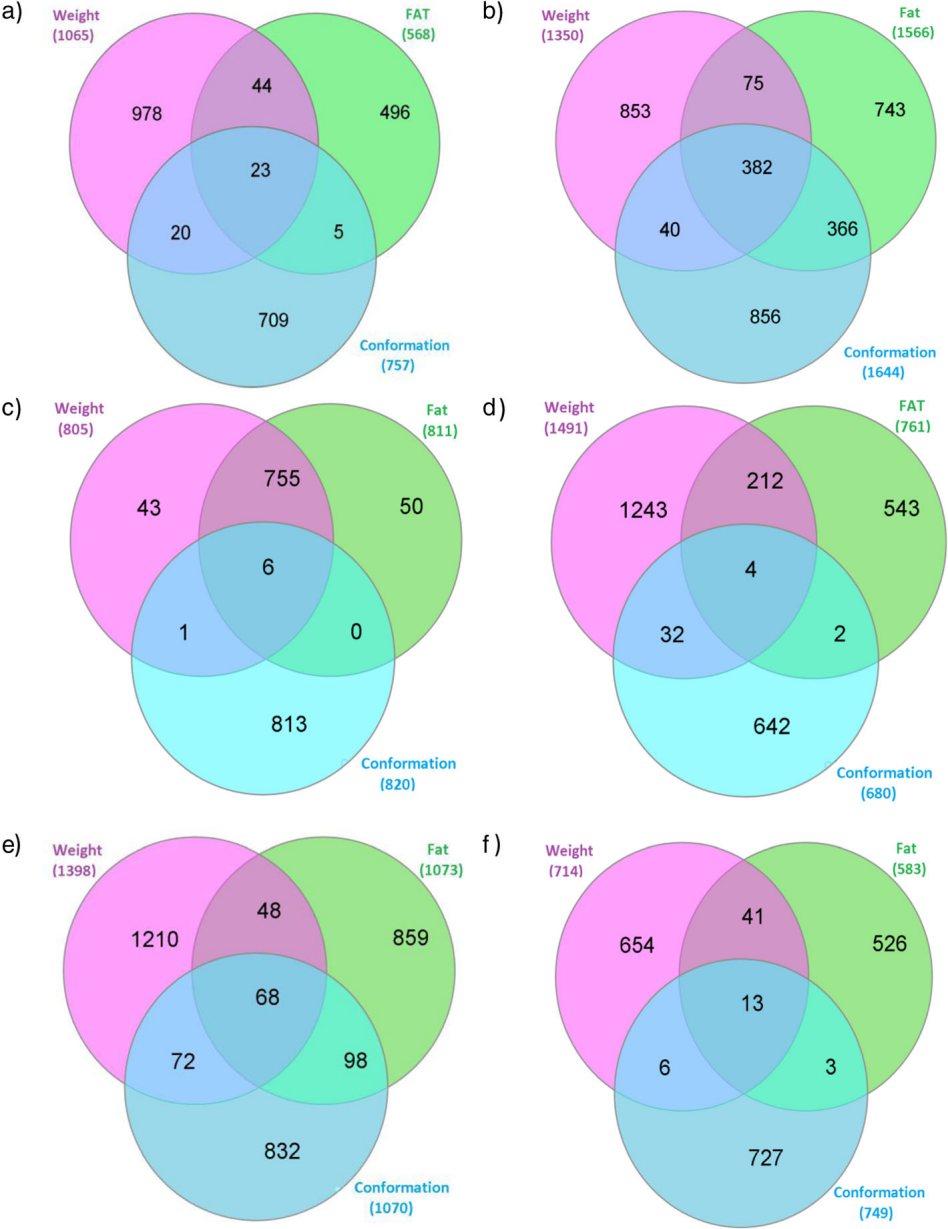
The number of significant 10 kb windows that overlapped across traits within each breed. Each window had to contain a SNP with an unadjusted p-value < 10^− 4^. **a** Angus **b**) Charolais **c**) Hereford **d**) Holstein-Friesian **e**) Limousin and **f**) Simmental. Pink represents carcass weight, green represents carcass fat and blue represents carcass conformation

Genomic regions associated with more than one carcass trait were identified using a similar approach to that used to detect associations across breeds; the genome was split into 10 kb windows and all significant windows that contained a SNP with an unadjusted *p*-value < 10^− 4^ were compared within breed across the three carcass traits. The degree of overlap across traits differed per breed, ranging from four (Holstein-Friesian) significant windows to 382 (Charolais) significant windows associated with carcass weight, fat and conformation (Fig. 6).

The least amount of overlap across all traits was within the HF population where only 4 windows on BTA22, 23 and 24 associated with carcass weight, fat and conformation (Fig. 6). Upstream variants of *EIF1B* were located in the window on BTA22, *ABCF1* and *PRRR3* were located within the window on BTA23, and the window on BTA24 was 160 kb downstream of *DOK6*. The same effect direction across traits was detected for all significant variants within the four windows. Limited overlap was also detected with the HE population (Fig. 4), although this may be a reflection of the smaller sample size within this breed. The CH population had the highest number of overlapping regions across all traits with 382 significant windows on BTA2 from 0.15 to 10.08 Mb, associated with carcass weight, fat and conformation. Overlapping regions across all three traits within the AA and LM populations were also located on BTA2 (AA 7.43 to 7.49 Mb; LM 3.16 to 10.07 Mb), with additional overlap located on BTA6 (AA 38.25 to 39.08 Mb; LM 40.16 to 40.18 Mb). The overlapping windows with the SI population were all located on BTA14 (from 26.15 to 26.38 Mb) and encompassed three genes; *ENSBTAG00000047136, UBXN2B* and *CYP7A1.*

A greater percentage of overlap was detected between carcass fat and conformation in the CH and LM populations, while in the remaining breeds a higher percentage of overlap was detected between carcass weight and conformation. A total of five genomic regions were identified on BTA2, 3, 17, 19, and 26 from significant windows shared just between carcass fat and conformation within the LM population; 85.71% of these 98 shared windows (Fig. 4) were located on BTA2 surrounding *MSTN* (from 0.95 to 10.09 Mb), no genes were identified within the windows on BTA3 and 17, and *GRB2* and *HSPA12A* were located within the windows on BTA19 and 26, respectively. Similarly to the LM population, 86.06% of the significant windows between carcass fat and conformation in the CH population were located on BTA2 from 0.00 Mb to 10.09 Mb; the remainder were located further downstream on BTA2 (from 21.68 to 25.68 Mb) as well as BTA17 (from 50.43 to 50.44 Mb and 66.47 to 66.49 Mb), BTA21 (from 47.98 to 47.99 Mb) and BTA28 (from 14.34 to 14.35 Mb).

Opposite SNP effect directions across traits were common; the majority of SNPs with an unadjusted *p*-value ≤10^− 4^ for carcass weight and conformation had an opposite SNP effect direction on carcass fat (Table 2). For example in the LM population, 95.33% of SNPs with a p-value ≤10^− 4^ for carcass weight differed in SNP effect direction for carcass fat. This trend was observed across all breeds with the exception of the HE population.

**Table 2:** The percentage of SNPs for each trait with an unadjusted *p*-value < 10^− 4^ within one carcass trait that differed in allele substitution effect in one of the two remaining carcass traits across six different breeds*

Trait	Opposing trait	AA	CH	HE	HF	LM	SI
Carcass weight	Carcass fat	87.03	91.58	2.86	39.34	95.33	74.43
	Carcass conformation	45.22	3.62	2.32	25.60	6.57	0.98
Carcass fat	Carcass weight	79.35	92.86	5.26	43.98	82.97	76.66
	Carcass conformation	74.58	91.97	2.47	25.85	90.31	75.09
Carcass conformation	Carcass weight	4.65	0.05	5.31	24.96	1.67	0.31
	Carcass fat	85.38	99.55	6.78	23.39	94.07	79.89

*SNPs with a *p*-value < 10^−4^ did not have to be significant in the comparison trait. Opposing trait represents the trait in which the SNPs effect directions where compared against. AA represents Angus, CH is Charolais, HE is Hereford, HF is Holstein-Friesian, LM is Limousin and SI is Simmental

### Pathway analysis

KEGG pathway analysis was completed within each breed for each trait and only pathways with an unadjusted p-value ≤0.05 are described in Additional file 4. The metabolic pathways identified in the HE population for carcass conformation contained the highest number of genes; 19 genes containing SNPs with an unadjusted p-value < 10^− 4^ were assigned to this pathway classification. Insulin related pathways were associated with carcass fat in 3 breeds (AA, CH and HF) (Additional file 4), whilst phosphorylation signalling related pathways were associated with carcass weight in CH, HF and SI populations. However, only the platelet activation pathway associated with carcass conformation in the AA population remained significant after Benjamini-Hochberg correction (adjusted *p* = 7.68 × 10^− 4^) and contained a total of 9 genes.

## Discussion

The ability to accurately identify when an animal may reach the desired carcass weight, subcutaneous fat level, and carcass conformation is desirable both from an economical and precision management perspective. Substantial genetic variability in the growth trajectories of young cattle has been previously reported by Englishby et al. [1], and suggests that management decisions such as penning of animals with expected similar growth patterns based on their genetic profile is feasible. Therefore, incorporating the genetic growth profile of an animal into decision support tools will enable more accurate bench-marking of herd profitability and possibly identify underperforming animals that may warrant further investigation. Additionally, by identifying the predisposing genomic factors that regulate growth and carcass traits, targeted nutritional supplements could be incorporated into an animal’s diet to enhance performance, where necessary. These developments facilitate increased animal and herd performance while possibly also reducing their environmental footprint. In the present study, we have successfully elucidated the genomic variation in three carcass performance trait that exists within and across six main cattle breeds. While major genes previously associated with carcass performance were confirmed, of particular interest was the discovery of several putative novel candidate genes that likely operate both within and across breeds. The majority of the associations detected in the present study were unique to each breed and this has implications for across breed genomic evaluations [14]. It is, however, important to note that the analyses was completed using imputed whole genome sequence (WGS) which may contain errors [15]; as such a precaution was taken to remove rare variants with a minor allele frequency (MAF) below 0.2% and regions of high Mendelian error rate, as detailed in the methods.

### Reaffirmation of known candidate genes

The role of myostatin on carcass merit for carcass traits has long since been established [16–18]. In the present study, QTL regions on BTA2 containing *MSTN* were associated with all three carcass traits in the CH and LM populations and with carcass conformation in the AA population. The Q204X mutation (rs110344317), a stop-gain disruption within the myostatin gene, although not the strongest association within each of the QTLs, did explain up to 6% of the genetic variance in each of the carcass traits in the CH population (carcass weight 1.21%; carcass fat 1.11% and carcass conformation 5.95%). Allais et al. [16] previously demonstrated that bulls carrying one copy of the Q204X mutation (i.e., the T allele) had greater carcass yields and conformation, concurrent with reduced intramuscular fat relative to non-carriers. A similar trend was seen in the present study; heterozygous carriers in the CH and LM populations had greater mean carcass weight and conformation estimated breeding values (EBVs) (CH mean carcass weight EBV 32.26, s.d 6.86; LM mean carcass weight EBV 25.07, s.d 6.62) than non-carriers of the Q204X mutation (CH mean carcass weight EBV 29.54, s.d 7.36; LM mean carcass weight EBV 20.52, s.d 6.75). However, this trend was not observed in the remaining breeds as the Q204X mutation was only marginally segregating in the AA and HE populations (MAF < 0.004), was under the MAF threshold for analyses in the SI population (MAF < 0.001) and was therefore not included in the analysis, and was monomorphic in the HF population; in comparison, the allele frequency of the Q204X mutation in the CH and LM populations was 0.11 and 0.03, respectively. Another *MSTN* variant which has also been shown to have a moderate muscle hypertrophy effect is the F94L mutation [19]. Although the F94L was not the strongest association for carcass related traits within the QTL on BTA2, it was significantly associated with carcass fat (unadjusted *p* = 5.99 × 10^− 10^) and conformation (unadjusted *p* = 9.61 × 10^− 13^) in the CH population in the present study; this was again reflected in the mean EBVs of homozygous carriers (*n* = 150) which had a greater mean EBV for carcass conformation (mean EBV 1.99, s.d 0.22) and reduced fat (mean EBV − 0.52, s.d 0.24) than non-carriers (conformation mean EBV 1.8, s.d 0.26; fat mean EBV − 0.31, s.d 0.27). The 150 homozygous carriers of the F94L mutation did not carry the Q204X mutation. Although the F94L mutation has been previously associated with increased carcass performance in the LM breed, this mutation was only significantly associated with carcass performance in the CH population in the present study due to poor imputation of the F94L SNP in the LM population (minimac r^2^ = 0.04). Similar to the Q204 mutation, the F94L mutation was only marginally segregating in the remaining beef breeds; this however still suggests that the targeted selection of the F94L and Q204X mutations for improved carcass performance through exploitation of the muscling hypertrophy phenotype within these breeds is feasible. Nevertheless, it is important to note that animals with the hypertrophy phenotype, on average, experience increased calving difficulty and reduced fertility [20], therefore many producers tend to avoid the mutations despite the increased beef production. Exploitation of the F94L mutation however, would enable producers to increase their carcass performance without possible adverse effects such as increased calving difficulty [18].

The strong known correlation between carcass weight and body size [21] implies that genomic regions and candidate genes previously associated with cattle height should share some associations with carcass weight, consistent with that observed in the present study in AA, CH and LM. In particular, QTL regions on BTA6 which contains the *NCAPG-LCORL* locus were associated with carcass weight in multiple breeds in the current study, corroborating results elsewhere in cattle studies [22–24]. Identifying which of these two genes is the causal gene has not been previously possible due to their close genomic proximity and subsequent strong LD patterns in the region [12]. In the present study, only three missense SNPs within the *NCAPG-LCORL* locus had a MAF > 0.002 in each of the AA, CH and LM populations and only two (rs109570900 and rs110251642) within NCAPG were of moderate significance in the AA population (unadjusted *p*-value = 7.65 × 10^− 5^ and 6.78 × 10^− 4^, respectively) while none were significant in the CH and LM populations; therefore it was not possible to infer the causative gene or mutation. While the strongest associations within the AA, CH, and LM populations were upstream of the *LCORL* gene, it is most likely that these associations are within enhancer regions of the *NCAPG/LCORL* complex and it is the expression quantity of the *NCAPG/LCORL* complex that is influencing carcass weight rather than a disruptive loss-of-function mutation.

To further elucidate the genomic overlap between carcass weight and stature, we examined the 163 SNPs variants that were recently identified by Bouwman et al. [12], to explain 13.8% of the phenotypic variation in cattle stature in a multi-breed population. Of these 163 variants, between 128 and 132 had a MAF ≥ 0.002 in each of our populations, but only two of the variants (rs109815800 and rs109676906) were associated (unadjusted *p*-value < 10^− 4^) with carcass weight in the HF population. Complete concordance however was not expected since the genetic correlation between height and carcass weight is just 0.69 (S.E. ± 0.06) [21]. The intronic variant rs109676906 located in *CCND2* on BTA5, has also been previously associated with height and insulin secretion in humans [25], whereas rs109815800, an intergenic variant, was located 6 kb downstream of *PLAG1*, a gene that has been well documented to be associated with stature in both humans [26] and cattle [27–29]. Indeed, the strongest association with carcass weight on BTA14 in the HF population in the present study was a downstream variant of *PLAG1*, suggesting that carcass weight may be influenced by the expression quantity of *PLAG1*, a hypothesis also supported by Karim et al. [27].

### Novel candidate genes

Within the AA population, a strong association for carcass weight was detected within 160 kb from the *ZFAT* gene on BTA14. *ZFAT*, which was the closest gene to the strongest associated SNP, has been previously associated with stature in both humans [30, 31] and horses [32, 33], and milking speed in French Holstein cows [34], but most interestingly is the fact that it was identified as the likely candidate gene within a lethal recessive haplotype detected in the AA population in Ireland [35]. The putatively lethal haplotype occurred at a frequency of 15.2% in the Irish AA population [35] and was also shown to be positively associated with weight-related traits and feed intake, thus providing further evidence for *ZFAT* as a likely breed-specific candidate gene for carcass weight within the AA population. Further work is needed on understanding exactly how this zinc finger is involved in carcass weight but its role in the development of the hematopoieic system [36] may be central as the hematopoietic system has been shown to be modulated by obesity [37, 38]. In the present study, only intronic and downstream gene variants within *ZFAT* were moderately significant (unadjusted *p*-value < 10^− 4^) and only two missense variants within *ZFAT* were segregating (rs483021047 and rs526028162), neither of which were significant in the AA population. As the strongest associations were located upstream of *ZFAT*, it is plausible that perhaps a regulatory region altering the expression of the zinc finger may be what is impacting its association with carcass weight. In addition, the gene *TMPRSS11A* was also identified as an AA-breed specific candidate association. Although, the role of *TMPRSS11A*, a transmembrane serine protease, in carcass weight is unclear, it was previously identified within a QTL associated with marbling score in Korean cattle [39]. The identification of rs452419999, a significant missense variant with a SIFT score of zero within TMPRSS11A suggests that possibly the loss-of-function of this gene is affecting carcass weight in AA.

Although the *MSTN* gene has been shown to associated with fat deposition [40], it is also plausible that other functional candidate genes within the QTL on BTA2 from 0.007 to 10.095 Mb also contribute to the carcass fat phenotype; exploitation of sequence information is invaluable to determining this. One such likely functional candidate gene is *SLC40A1* which makes the protein ferroportin and is involved in iron absorption [41]. The intronic SNP rs134895583, located near the start position of *SLC40A1* exhibited the second strongest association for carcass fat in the CH population (unadjusted *p*-value = 4.4 × 10^− 32^) and further evidence was provided when a genomic window 16 kb upstream of SLC40A1 was one of the four overlapping windows identified between the AA, CH and LM populations for carcass fat (Fig. 2b). No significant missense variant within *SLC40A1* was identified in the present study, although two 3′ UTR variants, rs209825163 and rs38033761, were associated with carcass fat in the CH population (unadjusted p-value = 1.33 × 10^− 13^ and 1.35 × 10^− 5^, respectively). Increased body fatness in humans has been associated with increased expression of *SLC40A1* [42] whereas in cattle lipogenic activities have been shown to be affected by iron content [43].

Olfactory receptors and the olfactory transduction pathway have been previously associated with feed intake in both cattle [44, 45] and pigs [46]. Although the mechanism of how olfactory receptors stimulate feed intake are unclear, recent evidence suggests that the endocannabinoid system drives eating behaviour by increasing odor detection [47, 48]. In the present study, a QTL on BTA15 containing multiple olfactory genes was associated with both carcass weight and fat in the CH population and with carcass fat in the multi-breed analysis. Although no missense variants were associated with both carcass weight and fat in the CH population, the olfactory receptor *ENSBTAG00000035988*, whose human ortholog is *OR8K3*, contained six significant downstream variants that were associated with both carcass weight and fat in the CH population and with carcass fat in the multi-breed analyses. This suggests that perhaps the expression quantities of *ENSBTAG00000035988* is influencing carcass performance; the allele frequencies of the positive alleles in the downstream variants ranged from 0.27 to 0.56 within each of the six breeds analysed suggesting targeted selection is indeed feasible. Another candidate gene identified located further upstream on BTA15 associated with carcass performance was *PRDM11*. A QTL containing *PRDM11* was associated with carcass weight in the multi-breed analysis and with both carcass weight and fat in the CH population. Although its role in carcass performance is unknown, a family member *PRDM16* has been previously documented as a “master regulator” of brown adipocyte differentiation and has been associated with improved metabolic phenotypes in mice [49].

Additional novel candidate genes identified from the multi-breed carcass analyses included *SORCS1*, *ARL15* and *MTCP2*. *SORCS1,* which was associated with carcass weight in the present study, has been previously associated with obesity induced type 2 diabetes mellitus [50], as well as being implicated as a receptor in the central control of energy balance [51]. Loss of both *SORCS1* and *SORCS3* in knockout mice resulted in greater food intake, decreased locomotor activity, and increased adiposity [51]. The most significant variant in *SORCS1,* rs4210220 an intronic variant, explained 0.35% of the genetic variance in the multi-breed analyses in the present study and was moderately segregating within each breed; the allele frequency of the positive allele ranged from 0.12 in HF to 0.47 in CH. *ARL15,* which was associated with carcass conformation in the present study, and is expressed in insulin responsive tissues such as adipose tissue and skeletal muscle, has been previously documented to regulate circulating levels of adiponectin in humans [52]. Reduced circulating levels of adiponectin, a protein hormone involved in regulating glucose and fatty acid breakdown, has been detected in obese humans and has been associated with insulin resistance in animal studies [53]. Lastly *MTCP2* on BTA21 which was associated with carcass fat in the present study, has also been previously associated with body fat and abdominal fat in humans [54] and with metabolic weight in Angus cattle [55].

Although plausible novel candidate genes were identified in the present study, the proportion of variance explained by these candidates was minimal thus reaffirming that although carcass performance may be affected by major genes in the form of *MSTN* and *NCAPG/LCORL*, the majority of variance is attributed to the additive (and possibly multiplicative) effect of many polymorphisms of small effect. In addition, the enrichment in the present study for non-coding variants (Table 1) suggests that carcass performance is influenced by regulatory variations that affect the expression quantity of identified candidate genes than through loss-of-function variants.

### Breaking correlations

Pleiotropy is thought to be one of the main causes of genetic correlations between traits [56] although linkage is also a contributing factor. A positive genetic correlation suggests that there may be shared QTL that effect both traits in the same direction, whereas a negative correlation suggests the existence of shared QTL that effect the traits in opposite directions. Identifying QTL with different patterns of linkage should help us to understand the physiological control of multiple traits [56]. For example, it is hoped that by identifying the QTL underlying carcass performance, the alleles underlying the antagonistic relationship between carcass fat and both carcass conformation and weight [1] could be resolved. Targeted selection of these alleles may facilitate increased selection for leaner, heavier animals that maintain a well-conformed carcass.

As the strongest SNP association may vary by trait, the present study used 10 kb windows to identify genomic regions associated with all three carcass traits. Genomic regions associated with all traits were identified in each of the breeds, although the majority of these regions were concentrated on BTA2 and BTA6, surrounding the major genes *MSTN* and *NCAPG/LCORL*, respectively. In the Charolais population, 56 SNPs located within and 5 kb up/downstream of *MSTN* and with a *p*-value < 10^− 4^ were associated with all three carcass traits and each SNP effect increased carcass weight and conformation whilst reducing carcass fat. This is in agreement with the literature which states that animals with hypertrophy are characterised as lean with low intramuscular fat content [40]. The negative correlation between carcass fat and both carcass weight and conformation is also reflected in the allele effect substitution directions; SNPs with a *p*-value < 10^− 4^ for carcass weight and conformation often increased carcass weight and conformation whilst decreasing carcass fat (Table 2). This trend is in agreement with the Irish beef breeding objectives which select heavier, leaner carcasses with better conformation.

As genes that operate in the same pathway might be expected to show the same pattern of effects [56], it was hoped pathways associated with all three carcass traits could be identified. Indeed only one pathway, the ECM-receptor interaction pathway in the LM population was associated with both carcass weight and conformation. This suggests that although major genes may be associated with all three carcass traits, the majority of genes containing significant variants (p-value < 10^− 4^) may be trait specific associations of small effect. However, it is important to note that the present study may not have been sufficiently powered to detect pleiotropic genes of larger effect.

## Conclusion

Our results reveal that the genetic architecture of carcass performance is highly polygenic across all six breeds. Although the role of major genes such as *MSTN* and *NCAPG/LCORL* on carcass performance were re-affirmed in the present study, the proportion of variance accounted for these major genes was minimal and rather it is the effect of many breed specific polymorphisms of small effect that are attributing to the genetic variation underlying carcass weight, fat and conformation. Indeed, only a few significant genomic regions were common across the large breed populations investigated in the present study and those identified were mainly centred around the *MSTN* and *NCAPG/LCORL* major gene complexes. Nevertheless, some candidate genes such as *SORCS1*, *MCTP2* and *ARL15* that exhibited associations with carcass merit in multiple breeds were identified, indicating that a portion of the genomic variation attributed to carcass merit is common across breeds which may have implications for across-breed genomic evaluations. Lastly, only a small proportion of significant genomic regions were shared across all three carcass traits emphasising that the majority of significant variants (unadjusted *p* < 10^− 4^) identified in the present study were not only breed specific but also trait specific.

## Methods

The data used in the present study originated from a pre-existing database managed by the Irish Cattle Breeding Federation (ICBF). Therefore, it was not necessary to obtain animal care and use committee approval in advance of conducting this study.

### Phenotypes

Cattle carcass weight in Ireland is measured, on average, 2 h after slaughter following the removal of the head, legs, thoracic and abdominal organs, internal fats, and hide. Carcass conformation and carcass fat grade are scored on the 15-point EUROP classification system from a video image analysis of each carcass; a carcass conformation score of 1 and a carcass fat score of 1 represents a poorly conformed carcass with little fat cover while and a carcass conformation score of 15 and a carcass fat score of 15 represents an excellently conformed carcass with considerable fat cover [1].

Estimated breeding values (EBVs) for carcass weight, carcass conformation and carcass fat and their associated reliabilities were obtained from the ICBF database from the December 2017 national genetic evaluation for all dairy and beef bulls. In Ireland, genetic evaluations for carcass traits are estimated using a multi-trait, multi-breed mixed model. Heritability estimates used in the national genetic evaluations were 40% for carcass weight, 35% for carcass conformation and 32% for carcass fat. Carcass phenotypes on 6,360,190 animals were included in the genetic evaluation with an associated pedigree file of 14,785,918 animals.

Of the animals with EBVs, only purebred (i.e. ≥87.5% of a single breed) genotyped sires with ≥5 carcass weight progeny records for any of the following breeds were retained for analysis; AA, CH, HE, HF, LM and SI. The effective record contribution (ERC) of each sire, taking into consideration what animals were genotyped, was estimated using the Harris and Johnston [57] method and only animals with an ERC ≥1 were retained for analysis. Deregression of the EBVs was completed using the secant method with a full animal model pedigree file. After edits, 28,470 sires from the six breeds were available for analysis which included 2366 AA, 11,219 CH, 1216 HE, 2372 HF, 9747 LM, and 1550 SI sires. The median ERC for carcass weight, conformation and fat was 6.93, 6.73 and 6.58, respectively.

### Genotype data

All 28,470 sires with carcass phenotypes were imputed to whole genome sequence as part of a larger dataset of 638,662 genotyped animals from multiple breeds. Each of the 28,470 sires included in the present study were genotyped on a variety of genotyping panels including the Illumina Bovine SNP50 (*n* = 717; 54,001 SNPs), Illumina High Density (HD; *n* = 3514; 777,962 SNPs), or the custom Irish Dairy and Beef (IDB) V1 (*n* = 3401; 16,622 SNPs), IDBV2 (*n* = 19,206; 16,223 SNPs) or IDBV3 (*n* = 2837; 52,445 SNPs) genotype panels. Prior to imputation to whole genome sequence (WGS), each of the 638,662 genotyped animals had a call rate ≥ 90% and only autosomal SNPs, SNPs with a known chromosome and position, and SNPs with a call rate ≥ 90% were retained within each panel.

All genotyped animals of the larger dataset were first imputed to HD using a two-step approach in FImpute2 [58]; this involved imputing the IDB-genotyped animals to the Bovine SNP50 density and subsequently imputing all resulting genotypes, including the Bovine SNP50 genotypes, to HD using a multi-breed reference population of 5504 HD genotyped animals. Imputation of all 638,662 HD imputed animals to WGS was then undertaken using a reference population of 2333 *Bos Taurus* animals of multiple breeds from Run6.0 of the 1000 Bulls Genomes Project. All variants in the reference population were called using SAMtools and genotype calls were improved using Beagle software to provide a consensus SNP density across all animals. Details of alignment to UMD 3.1, variant calling and quality controls completed within the multi-breed population are described by Daetwyler et al. [15] for a subset of the animals. In total, 41.39 million SNP variants were identified across the genome and the average coverage was 12.85X. Imputation of the HD genotypes to WGS was achieved by firstly phasing all 638,662 HD imputed animals using Eagle ([59]; version 2.3.2)) and subsequently imputing all animals to WGS using minimac3 [60]. To quantify the accuracy of imputation to WGS, a validation set was constructed which consisted of 175 sequenced animals that had also been genotyped on either the Bovine SNP50 or HD genotype panel. Validation involved imputing the animal’s genotypes to WGS using the aforementioned approach minus the sequence data of the 175 validation animals in the reference population. The average genotype concordance across all SNPs, defined as the proportion of correctly called genotypes, was estimated to be 0.98.

Regions with possible poor WGS imputation accuracy were identified using a dataset of 147,309 verified parent-progeny relationships from the 638,662 genotyped dataset; such poor imputation could perhaps be due to local mis-assemblies or mis-orientated contigs. Mendelian errors, defined as the proportion of opposing homozygotes in a parent-progeny pair, were estimated for each relationship and the subsequent Mendelian error rate per SNP was determined. To accurately identify genomic regions of poor imputation, the R package GenWin [61] which fits a β-spline to the data to find likely inflection points, was used to identify genomic region breakpoints with high Mendelian errors. Windows were analyzed using an initial window size of 5 kb and Genwin pooled windows for which the SNP Mendelian error rate were similar. The average SNP Mendelian error rate per window was estimated and all variants within windows where the mean SNP Mendelian error rate was > 0.02 were removed; a total of 687,137 SNPs were discarded.

To further refine the WGS imputed dataset consisting of 28,470 sires with genotype and EBV information in the present study, all SNPs with a MAF < 0.002 across all animals were removed for the multi-breed analysis, and SNPs with a MAF < 0.002 within each breed were removed for the within-breed analysis. Following edits, 18,863,675 imputed SNPs remained for analysis across all breeds and 16,657,735, 17,945,687, 16,916,637, 15,409,084, 18,029,324, and 17,890,329 imputed SNPs remained within the AA, CH, HE, HF, LM and SI breeds, respectively. The average minimac r^2^ across all SNPs was 0.81.

### Genome-wide association analyses

Whole genome association analyses were performed within each breed separately, as well as in a dataset of all breeds combined, using an animal linear mixed model in Wombat [62]. To account for population relatedness, a genomic relationship matrix among all animals was constructed using Method I of the VanRaden [63] based on just the imputed autosomal SNPs from the edited HD panel (*n* = 642,153 SNPs). All imputed sequence SNPs, scored as 0, 1 or 2, were included individually as a fixed effect covariate in the model one at a time. The equation for the whole genome association analysis is detailed below;

$$ y=\mu + bx+g+e $$

where y is the deregressed EBV, *μ* is the mean term, *b* is fixed effect of the candidate SNP to be tested for association, *x* is the vector of imputed genotypes*, g ~ N(0,G* ${\upsigma}_{\mathrm{u}}^2$) is the vector of additive genetic effects, where *G* is the genomic relationship matrix calculated from the HD SNP genotypes, and ${\upsigma}_{\mathrm{u}}^2$ is the additive genetic variance, and e ~ N(0, ***I***${\upsigma}_{\mathrm{e}}^2$) is the vector of random residual effects, and ***I***${\upsigma}_{\mathrm{e}}^2$ is the residual variance. Breed was included as a fixed effect for the multi-breed analyses. Each dependent variable was also weighted using the approach outlined by Garrick et al., [64];

$$ {w}_i=\frac{1-{h}^2}{\left[c+\frac{1-{r}_i^2}{r_i^2}\right]{h}^2} $$

where *w*_*i*_ is the weighting factor of the *i*th deregressed EBV, *h*^2^ is the heritability estimate for each carcass trait, ${r}_i^2$ is the reliability of the *i*th deregressed EBV and c is the proportion of genetic variance not accounted by the SNPs and set at 0.9 for analyses thus allowing each SNP to attribute up to 10% of the genetic variance. Test statistics for all SNPs were obtained and converted into their corresponding *p*-values. The genomic inflation factor was estimated and ranged from 0.98 in the AA population to 1.02 in the LM population; the multi-breed GWAS did exhibit inflation (λ = 1.28) and as such the p-values from the multi-breed analyses were adjusted accordingly. The Benjamini and Hochberg method assuming a false discovery rate of 5% was used to correct for multiple testing; the number of tests assumed was equal to the number of SNPs across the entire genome and differed slightly per breed from 15,409,084 to 18,863,675. The proportion of the genetic variance in each carcass trait attributable to individual SNPs was calculated as 2*pqa*^2^/*σ*^*2*^, where *p* was the major allele frequency, *q* was the minor allele frequency, *a* was the estimated allele substitution effect and *σ*^*2*^ was the genetic variance for the phenotype under investigation.

### Defining QTL

Within each of the analyses, QTL regions were defined as all regions where a minimum of three SNPs were significantly associated with the trait of interest following adjustment for multiple testing using the Benjamini and Hochberg approach with a false discovery rate of 5%, and that resided within 500 kb of each other. Genes within and overlapping each QTL were identified using Ensembl (http://ensemble.org) and NCBI map viewer (http://www.ncbi.nlm.nih.gov/mapview) on the bovine UMD 3.1. Candidate genes were chosen from QTL based on previous literature and their biological function. If no gene resided in the QTL region, genes within 250 kb of the start and end position of the QTL, were considered as putative candidate genes. Previously reported cattle QTL were obtained from the animal QTLdb (http://www.animalgenome.org/cgi-bin/QTLdb/index).

To identify QTL present in more than one breed, each chromosome was split into 10 kb windows and each window that contained a SNP with a non-adjusted *p*-value < 10^− 4^ present in two or more breeds, was considered a putative across-breed QTL. A similar approach was used to detect QTL common to the three carcass traits. This threshold was previously applied by Tenghe et al., [65] when detecting across trait QTLs and allows for putative across breed and across trait regions to be identified with less stringency.

### Pathway analysis

To identify over-represented pathways associated with carcass performance within each breed, all genes containing SNPs with a non-adjusted p-value < 10^− 4^ for carcass weight, fat and conformation within each breed were analysed using the Database for Annotation, Visualization and Integrated Discovery (DAVID) v.6.8. *P*-values were calculated by EASE (an adoption of the Fisher Exact test to measure the gene-enrichment in annotation terms) and Benjamini-Hochberg was used to correct for multiple testing.

## Additional files

Additional file 1: Quantitative trait loci (QTL) associated with carcass weight. All QTL significantly associated with carcass weight within six breeds after adjustment for Benjamini and Hochberg multiple testing. (DOCX 37 kb)

Additional file 2: Quantitative trait loci (QTL) associated with carcass fat. All QTL significantly associated with carcass fat within six breeds after adjustment for Benjamini and Hochberg multiple testing. (DOCX 27 kb)

Additional file 3: Quantitative trait loci (QTL) associated with carcass conformation. All QTL significantly associated with carcass conformation within six breeds after adjustment for Benjamini and Hochberg multiple testing. (DOCX 29 kb)

Additional file 4: Significant pathways (*p* value <0.05) associated with carcass weight, fat and conformation in six breeds. All KEGG pathways associated with carcass weight, fat and conformation identified from using all genes containing a SNP with an unadjusted *p*-value of < 10^− 4^ from each of the respective association analyses in each breed. (DOCX 24 kb)

## Data Availability

Sequence variant genotypes were provided by participation in the 1000 Bulls consortium and can be found at NCBI BioProject PRJNA238491, PRJEB9343, PRJNA176557, PRJEB18113, PRNJA343262, PRJNA324822, PRJNA324270, PRJNA277147, PRJNA474946 and PRJEB5462. For the remaining sequences the board of the 1000 Bull Genome Consortium should be contacted. Individual genotype and phenotype data used in this study is also managed by a third party, the Irish Cattle Breeding Federation. Requests for genotype data can be made to the Irish Cattle Breeding Federation, Highfield House, Shinagh, Bandon, Co. Cork, Ireland: email query@icbf.com; fax: + 353 (0)238820229; phone: + 353 (0)238820222; website: www.icbf.com. All significant associations identified in the present study are provided within the manuscript and through additional material.

## Abbreviations

AA: Angus
CH: Charolais
EBV: Estimated breeding value
ERC: Effective record contribution
GWAS: Genome-wide association study
HD: High density
HE: Hereford
HF: Holstein-Friesian
IDB: Irish dairy and beef
LM: Limousin
MAF: Minor allele frequency
QTL: Quantitative trait loci
SI: Simmental
SNP: Single nucleotide polymorphism
WGS: Whole genome sequence
