# Supplementary material for: Reaffirmation of known major genes and the identification of novel candidate genes associated with carcass-related metrics based on whole genome sequence within a large multi-breed cattle population
Source: BMC Genomics. 2019 Sep 18;20:720. doi: 10.1186/s12864-019-6071-9 (PMC6751660; doi:10.1186/s12864-019-6071-9)
Supplement: Supplementary file 2 — Quantitative trait loci (QTL) associated with carcass fat. All QTL significantly associated with carcass fat within six breeds after adjustment for Benjamini and Hochberg multiple testing. (DOCX 27 kb) [file 12864_2019_6071_MOESM2_ESM.docx]

**Additional File 2**: Quantitative trait loci (QTL) associated with carcass fat^1^. QTL were defined as genomic regions where 3 SNPs within 500kb of each other remained significant after adjustment for Benjamini and Hochberg correction.

|  |  |  |  |  |  |  |  |  | Allele Frequency of + Allele | | | | | |  |  |
| --- | --- | --- | --- | --- | --- | --- | --- | --- | --- | --- | --- | --- | --- | --- | --- | --- |
| Breed | Chr | Start | End | No Sig SNPs | Strongest SNP | P-value | Allele | Effect | AA | CH | HE | HF | LM | SI | No Genes | Candidate gene |
| AA | - | - | - | - | - | - | - | - | - | - | - | - | - | - | - | - |
| CH | 1 | 148.459 | 148.527 | 66 | 148,459,370^a^ | 2.73x10^-6^ | T | 0.026 | 0.113 | 0.211 | 0.079 | 0.266 | 0.181 | 0.112 | 59 | ENSBTAG00000046447 |
|  | 2 | 0.007 | 10.095 | 6512 | 6,701,015^a^ | 3.70x10^-32^ | A | 0.070 | 0.933 | 0.726 | 0.725 | 0.845 | 0.903 | 0.815 | - | MSTN/SLC40A1 |
|  | 2 | 11.888 | 11.902 | 12 | 11,902,411^a^ | 6.99x10^-6^ | G | 0.026 | 0.614 | 0.829 | 0.776 | 0.692 | 0.561 | 0.895 | - | ENSBTAG00000038064 |
|  | 2 | 21.690 | 22.595 | 24 | 21,710,487^a^ | 3.07x10^-8^ | C | 0.028 | 0.274 | 0.478 | 0.081 | 0.492 | 0.317 | 0.275 | 12 | ATP5G3 |
|  | 2 | 24.534 | 24.737 | 76 | 24,536,591^c^ | 9.81x10^-7^ | T | 0.027 | 0.519 | 0.599 | 0.589 | 0.554 | 0.583 | 0.556 | 3 | SLC25A12 |
|  | 2 | 25.645 | 25.680 | 69 | 25,646,335^d^ | 3.31x10^-8^ | C | 0.033 | 0.903 | 0.801 | 0.982 | 0.976 | 0.869 | 0.798 | 1 | GAD1^†^ |
|  | 2 | 34.384 | 34.785 | 8 | 34,785,255^c^ | 5.19x10^-6^ | T | 0.051 | 0.077 | 0.051 | 0.016 | 0.049 | 0.006 | 0.004 | 4 | ENSBTAG00000048246 |
|  | 2 | 39.155 | 39.176 | 7 | 39,162,380^a^ | 3.69X10^-6^ | A | 0.087 | 0.992 | 0.988 | 0.995 | 0.981 | 0.997 | 0.996 | - | ACVR1 |
|  | 2 | 89.731 | 89.773 | 10 | 89,762,222^c^ | 8.95x10^-6^ | T | 0.024 | 0.264 | 0.479 | 0.427 | 0.318 | 0.324 | 0.403 | 1 | AOX4^†^ |
|  | 2 | 113.940 | 113.951 | 65 | 113,944,450^a^ | 1.92X10^-6^ | T | 0.027 | 0.432 | 0.367 | 0.429 | 0.292 | 0.409 | 0.264 | - | DOCK10 |
|  | 5 | 28.875 | 28.900 | 7 | 28,875,621^a^ | 1.30X10^-5^ | C | 0.026 | 0.722 | 0.712 | 0.523 | 0.532 | 0.812 | 0.687 | 1 | ENSBTAG00000032914/ LETMD1 |
|  | 5 | 32.072 | 32.151 | 23 | 32,132,049^a^ | 1.45x10^-7^ | C | 0.032 | 0.680 | 0.796 | 0.869 | 0.655 | 0.626 | 0.692 | 1 | ZNF641 |
|  | 5 | 51.418 | 51,566 | 5 | 51,565,657^a^ | 2.45x10^-6^ | T | 0.137 | 0.998 | 0.996 | 0.000 | 0.964 | 0.996 | 0.997 | 1 | USP15 |
|  | 6 | 27.740 | 27.761 | 8 | 27,742,297^a^ | 1.46x10^-6^ | A | 0.026 | 0.596 | 0.596 | 0.566 | 0.659 | 0.709 | 0.572 | - | RAP1GDS1 |
|  | 7 | 44.017 | 44.020 | 5 | 44,019,727^a^ | 5.86x10^-6^ | G | 0.034 | 0.016 | 0.137 | 0.051 | 0.159 | 0.015 | 0.036 | - | PGB2 |
|  | 8 | 83.575 | 83.699 | 39 | 83,652,278^a^ | 4.63x10^-7^ | G | 0.028 | 0.225 | 0.280 | 0.382 | 0.405 | 0.486 | 0.386 | 1 | ENSBTAG00000048213 |
|  | 9 | 82.048 | 82.056 | 6 | 82,048,095^a^ | 1.38x10^-8^ | A | 0.077 | 0.029 | 0.028 | 0.000 | 0.016 | 0.111 | 0.013 | - | FUCA2 |
|  | 11 | 31.004 | 31.022 | 8 | 31,021,908^a^ | 4.62x10^-6^ | C | 0.049 | 0.021 | 0.039 | 0.034 | 0.016 | 0.133 | 0.097 | - | ENSBTAG00000027015 |
|  | 14 | 63.395 | 63.402 | 4 | 63,395,051^e^ | 1.67x10^-6^ | G | 0.035 | 0.006 | 0.115 | 0.207 | 0.103 | 0.020 | 0.029 | 1 | FZD6^†^ |
|  | 15 | 34.932 | 37.962 | 121 | 37,912,248^c^ | 1.55x10^-6^ | T | 0.024 | 0.409 | 0.493 | 0.473 | 0.219 | 0.489 | 0.485 | 20 | SOX6 |
|  | 15 | 59.389 | 60.087 | 30 | 60,058,156^a^ | 5.6x10^-7^ | A | 0.155 | 0.944 | 0.996 | 0.961 | 0.997 | 0.996 | 0.993 | 2 | KIF18A |
|  | 15 | 73.757 | 74.103 | 7 | 73,761,064^a^ | 4.27x10^-7^ | A | 0.038 | 0.022 | 0.068 | 0.088 | 0.080 | 0.077 | 0.034 | - | API5 |
|  | 15 | 75.566 | 77.379 | 224 | 76,676,130^a^ | 3.50x10^-14^ | G | 0.045 | 0.513 | 0.648 | 0.681 | 0.539 | 0.479 | 0.71 | 18 | CRY2 |
|  | 15 | 78.305 | 82.375 | 319 | 78,401,390^fd^ | 1.17x10^-8^ | A | 0.032 | 0.379 | 0.456 | 0.423 | 0.336 | 0.429 | 0.318 | 130 | ENSBTAG00000035988^†^ |
|  | 16 | 16.540 | 16.580 | 110 | 16,568,520^a^ | 1.18x10^-6^ | T | 0.027 | 0.368 | 0.358 | 0.089 | 0.396 | 0.319 | 0.298 | - | - |
|  | 17 | 50.430 | 50.464 | 14 | 50,430,264^a^ | 5.78x10^-6^ | G | 0.039 | 0.949 | 0.912 | 0.750 | 0.852 | 0.915 | 0.967 | - | - |
|  | 17 | 66.201 | 66.983 | 263 | 66,807,225^a^ | 1.14x10^-8^ | A | 0.034 | 0.826 | 0.713 | 0.622 | 0.811 | 0.573 | 0.742 | 16 | WSCD2 |
|  | 20 | 66.508 | 66.515 | 8 | 66,514,494^a^ | 5.59x10^-7^ | C | 0.027 | 0.423 | 0.454 | 0.411 | 0.307 | 0.418 | 0.429 | - | TENT4A |
|  | 23 | 29.317 | 29.829 | 13 | 29,353,645^a^ | 2.21x10^-6^ | T | 0.024 | 0.552 | 0.630 | 0.755 | 0.809 | 0.544 | 0.661 | - | ENSBTAG00000037628 |
|  | 29 | 43.500 | 43.842 | 39 | 43,839,244^a^ | 3.29x10^-6^ | A | 0.025 | 0.581 | 0.531 | 0.667 | 0.698 | 0.571 | 0.546 | 16 | PP2R5B |
| HE | - | - | - | - | - | - | - | - | - | - | - | - | - | - | - | - |
| HF | - | - | - | - | - | - | - | - | - | - | - | - | - | - | - | - |
| LM | 2 | 4.916 | 12.118 | 510 | 7,492,224^a^ | 1.59x10^-17^ | G | 0.111 | 0.575 | 0.555 | 0.663 | 0.637 | 0.95 | 0.569 | 37 | MSTN/SLC40A1 |
|  | 2 | 105.665 | 105.668 | 3 | 105,666,691^a^ | 1.77x10^-6^ | T | 0.028 | 0.524 | 0.546 | 0.622 | 0.657 | 0.619 | 0.503 | - | IGFBP5 |
|  | 6 | 33.477 | 33.763 | 82 | 33,601,050^a^ | 4.38x10^-8^ | G | 0.060 | 0.940 | 0.944 | 0.958 | 0.872 | 0.929 | 0.958 | 1 | ENSBTAG00000047821 |
|  | 6 | 40.136 | 40.178 | 23 | 40,144,623^a^ | 3.93x10^-9^ | G | 0.096 | 0.890 | 0.815 | 0.961 | 0.890 | 0.979 | 0.876 | - | NCAPG/LCORL |
|  | 7 | 84.823 | 84.860 | 7 | 84,823,604^a^ | 1.25x10^-6^ | A | 0.169 | 0.023 | 0.060 | 0.119 | 0.076 | 0.004 | 0.073 | - | RPS23 |
|  | 8 | 27.682 | 27.750 | 59 | 27,708,892^a^ | 1.75x10^-12^ | T | 0.041 | 0.589 | 0.596 | 0.513 | 0.514 | 0.704 | 0.620 | - | BNC2 |
|  | 8 | 40.291 | 40.307 | 5 | 40,306,091^a^ | 2.17x10^-7^ | T | 0.030 | 0.209 | 0.283 | 0.147 | 0.316 | 0.309 | 0.368 | - | SLC1A1 |
|  | 14 | 26.150 | 26.327 | 27 | 26,320,235^a^ | 3.19x10^-7^ | T | 0.054 | 0.970 | 0.890 | 0.989 | 0.851 | 0.947 | 0.947 | 2 | UBXN2B |
|  | 19 | 56.759 | 56.819 | 6 | 56,760,409^c^ | 7.58x10^-7^ | T | 0.026 | 0.860 | 0.623 | 0.690 | 0.939 | 0.616 | 0.659 | 1 | GRB2^†^ |
| SI | - | - | - | - | - | - | - | - | - | - | - | - | - | - | - | - |

^1^BTA: Bos taurus autosome number. SNP: name of single nucleotide polymorphism. P-value: unadjusted p-value of SNP. No of genes is the number of genes in the QTL. If no gene was present in the QTL the nearest functional candidate gene within 250kb was chosen. AA represents Angus, CH is Charolais, He is Hereford, HF is Holstein-Friesian, LM is Limousin and SI is Simmental. ^a^intergenic, ^b^missense, ^c^intronic, ^d^downstream gene variant, ^e^upstream gene variant, ^f^5’ UTR, ^†^ gene in which the most significant SNP within the QTL was identified in. The Benjamini and Hochberg threshold with a false discovery rate of 5% was 2.29x10^-5^, and 2.02 X10^-6^ within the CH and LM populations.
