# Supplementary material for: Reaffirmation of known major genes and the identification of novel candidate genes associated with carcass-related metrics based on whole genome sequence within a large multi-breed cattle population
Source: BMC Genomics. 2019 Sep 18;20:720. doi: 10.1186/s12864-019-6071-9 (PMC6751660; doi:10.1186/s12864-019-6071-9)
Supplement: Supplementary file 4 — Significant pathways (p value <0.05) associated with carcass weight, fat and conformation in six breeds. All KEGG pathways associated with carcass weight, fat and conformation identified from using all genes containing a SNP with an unadjusted p-value of < 10− 4 from each of the respective association analyses in each breed. (DOCX 24 kb) [file 12864_2019_6071_MOESM4_ESM.docx]

**Additional File 4**. Significant pathways (p value <0.05) associated with carcass weight, fat and conformation in six breeds. AA represents Angus, CH represents Charolais, HE is Hereford, HF is Holstein-Friesian, LM is Limousin and SI is Simmental*

| Trait | Breed | No. SNPs | Ensembl IDS | Mapped genes | Term | Count | PValue | Benjamini |
| --- | --- | --- | --- | --- | --- | --- | --- | --- |
| Carcass weight | AA | 5049 |  |  | Prion diseases | 3 | 0.016 | 0.894 |
|  |  |  |  |  | Gap junction | 4 | 0.016 | 0.688 |
|  | CH | 6583 |  | 234 | Glutamatergic synapse | 6 | 0.015 | 0.910 |
|  |  |  |  | Platelet activation | 6 | 0.024 | 0.851 |  |
|  |  |  |  | Phosphatidylinositol signaling system | 5 | 0.038 | 0.866 |  |
|  | HE | 2372 | 166 | 145 | ECM-receptor interaction | 4 | 0.023 | 0.933 |
|  |  |  |  |  | Regulation of lipolysis in adipocytes | 3 | 0.056 | 0.967 |
|  | HF | 4110 | 284 | 243 | Cell cycle | 7 | 0.005 | 0.593 |
|  |  |  |  |  | Prolactin signaling pathway | 5 | 0.014 | 0.726 |
|  |  |  |  |  | Transcriptional misregulation in cancer | 7 | 0.023 | 0.752 |
|  |  |  |  |  | Chemokine signaling pathway | 7 | 0.030 | 0.744 |
|  |  |  |  |  | PI3K-Akt signaling pathway | 10 | 0.031 | 0.683 |
|  |  |  |  |  | cAMP signaling pathway | 7 | 0.039 | 0.703 |
|  |  |  |  |  | Endocytosis | 8 | 0.046 | 0.708 |
|  |  |  |  |  | Focal adhesion | 7 | 0.048 | 0.673 |
|  | LM | 7413 | 233 | 204 | ECM-receptor interaction | 6 | 0.002 | 0.242 |
|  | SI | 2226 | 163 | 137 | Thyroid hormone synthesis | 5 | 0.001 | 0.065 |
|  |  |  |  |  | cGMP-PKG signaling pathway | 6 | 0.002 | 0.127 |
|  |  |  |  |  | Vascular smooth muscle contraction | 5 | 0.005 | 0.160 |
|  |  |  |  |  | Platelet activation | 5 | 0.006 | 0.148 |
|  |  |  |  |  | Long-term potentiation | 4 | 0.006 | 0.124 |
|  |  |  |  |  | Rap1 signaling pathway | 6 | 0.007 | 0.123 |
|  |  |  |  |  | Oxytocin signaling pathway | 5 | 0.011 | 0.160 |
|  |  |  |  |  | Salivary secretion | 4 | 0.011 | 0.151 |
|  |  |  |  |  | Chemokine signaling pathway | 5 | 0.020 | 0.228 |
|  |  |  |  |  | Retrograde endocannabinoid signaling | 4 | 0.021 | 0.213 |
|  |  |  |  |  | Inflammatory mediator regulation of TRP channels | 4 | 0.021 | 0.213 |
|  |  |  |  |  | Calcium signaling pathway | 5 | 0.022 | 0.203 |
|  |  |  |  |  | Pathways in cancer | 7 | 0.024 | 0.207 |
|  |  |  |  |  | Glutamatergic synapse | 4 | 0.026 | 0.205 |
|  |  |  |  |  | Sphingolipid signaling pathway | 4 | 0.030 | 0.221 |
|  |  |  |  |  | Tight junction | 4 | 0.042 | 0.279 |
|  |  |  |  |  | Adrenergic signaling in cardiomyocytes | 4 | 0.048 | 0.294 |
|  |  |  |  |  |  |  |  |  |
| Carcass fat | AA | 1322 | 115 | 104 | Serotonergic synapse | 4 | 0.013 | 0.777 |
|  |  |  |  |  | Insulin secretion | 3 | 0.046 | 0.941 |
|  | CH | 11930 | 267 | 234 | Insulin secretion | 6 | 0.006 | 0.662 |
|  |  |  |  |  | Aldosterone synthesis and secretion | 5 | 0.025 | 0.904 |
|  |  |  |  |  | Glucagon signaling pathway | 5 | 0.045 | 0.940 |
|  | HE | 2510 | 163 | 143 | ECM-receptor interaction | 4 | 0.022 | 0.923 |
|  |  |  |  |  | Vascular smooth muscle contraction | 4 | 0.049 | 0.948 |
|  | HF | 2139 | 182 | 154 | Morphine addiction | 4 | 0.023 | 0.958 |
|  |  |  |  |  | ABC transporters | 3 | 0.031 | 0.883 |
|  |  |  |  |  | Type II diabetes mellitus | 3 | 0.038 | 0.829 |
|  | LM | 4975 | 236 | 214 | Ubiquitin mediated proteolysis | 7 | 0.003 | 0.441 |
|  | SI | 1915 | 150 | 131 | Biosynthesis of antibiotics | 5 | 0.031 | 0.964 |
|  |  |  |  |  |  |  |  |  |
| Carcass conformation | AA | 2278 | 179 | 162 | Platelet activation | 9 | 4.712x10^-6^ | 7.679x10^-4^ |
|  |  |  |  |  | Phosphatidylinositol signalling system | 6 | 0.001 | 0.068 |
|  |  |  |  |  | Gastric acid secretion | 5 | 0.002 | 0.111 |
|  |  |  |  |  | Focal adhesion | 7 | 0.005 | 0.181 |
|  |  |  |  |  | Estrogen signalling pathway | 5 | 0.007 | 0.192 |
|  |  |  |  |  | Inflammatory mediator regulation of TRP channels | 5 | 0.008 | 0.197 |
|  |  |  |  |  | cGMP-PKG signalling pathway | 6 | 0.009 | 0.185 |
|  |  |  |  |  | Thyroid hormone signalling pathway | 5 | 0.011 | 0.197 |
|  |  |  |  |  | Thyroid hormone synthesis | 4 | 0.016 | 0.247 |
|  |  |  |  |  | Salivary secretion | 4 | 0.025 | 0.342 |
|  |  |  |  |  | Oxytocin signalling pathway | 5 | 0.029 | 0.355 |
|  |  |  |  |  | Aldosterone-regulated sodium reabsorption | 3 | 0.036 | 0.388 |
|  | CH | 19873 | 197 | 170 | ECM-receptor interaction | 5 | 0.010 | 0.728 |
|  |  |  |  |  | Glutathione metabolism | 4 | 0.016 | 0.632 |
|  |  |  |  |  | Regulation of actin cytoskeleton | 7 | 0.017 | 0.526 |
|  |  |  |  |  | Drug metabolism - cytochrome P450 | 4 | 0.019 | 0.454 |
|  |  |  |  |  | Metabolism of xenobiotics by cytochrome P450 | 4 | 0.020 | 0.398 |
|  |  |  |  |  | Chemical carcinogenesis | 4 | 0.031 | 0.485 |
|  | HE | 2861 | 195 | 177 | Retrograde endocannabinoid signalling | 5 | 0.013 | 0.886 |
|  |  |  |  |  | Metabolic pathways | 19 | 0.020 | 0.814 |
|  |  |  |  |  | Sphingolipid signalling pathway | 5 | 0.021 | 0.689 |
|  |  |  |  |  | Axon guidance | 5 | 0.024 | 0.633 |
|  |  |  |  |  | Nucleotide excision repair | 3 | 0.060 | 0.866 |
|  | HF | 1795 | 166 | 149 | Tight junction | 5 | 0.023 | 0.944 |
|  | LM | 5141 | 182 | 161 | ECM-receptor interaction | 5 | 0.005 | 0.467 |
|  |  |  |  |  | Focal adhesion | 7 | 0.005 | 0.308 |
|  |  |  |  |  | Protein digestion and absorption | 4 | 0.027 | 0.720 |
|  | SI | 1930 | 175 | 154 | Fc gamma R-mediated phagocytosis | 4 | 0.020 | 0.951 |
|  |  |  |  |  | Bladder cancer | 3 | 0.030 | 0.897 |
|  |  |  |  |  | Non-small cell lung cancer | 3 | 0.056 | 0.941 |

*No of SNPs is the number of significant SNPs after Benjamini and Hochberg correction
